# The Protein Data Bank archive as an open data resource

**DOI:** 10.1007/s10822-014-9770-y

**Published:** 2014-07-26

**Authors:** Helen M. Berman, Gerard J. Kleywegt, Haruki Nakamura, John L. Markley

**Affiliations:** 1 RCSB PDB, Department of Chemistry and Chemical Biology and Center for Integrative Proteomics Research, Rutgers, The State University of New Jersey, Piscataway, NJ 08854 USA; 2 PDBe, European Molecular Biology Laboratory–European Bioinformatics Institute, Cambridge, CB10 1SD UK; 3 PDBj, Institute for Protein Research, Osaka University, 3-2 Yamadaoka, Suita, Osaka 565-0871 Japan; 4 BioMagResBank, Department of Biochemistry, University of Wisconsin-Madison, Madison, WI 53706 USA

**Keywords:** Protein Data Bank, Protein structure, Biomacromolecules, Data archive

## Abstract

The Protein Data Bank archive was established in 1971, and recently celebrated its 40th anniversary (Berman et al. in Structure 20:391, 2012). An analysis of interrelationships of the science, technology and community leads to further insights into how this resource evolved into one of the oldest and most widely used open-access data resources in biology.

## Early history of protein crystallography

In 1934, Dorothy Crowfoot (Hodgkin) together with John D. Bernal at Cambridge University obtained the first diffraction pattern of the protein pepsin [1]. Bernal had trained in crystallography at the Royal Institution in London with Sir William Henry Bragg, who with his son Sir William Lawrence Bragg founded the field of X-ray crystallography. Bernal went on to establish his own research group in Cambridge. He was a visionary figure in the field earning the nickname “Sage” while still an undergraduate at Cambridge. He had strong views about the interactions of science and society and felt that science had to be useful, in opposition to others who voiced that science should be pure and separated from societal needs [2]. His philosophies continue to influence how crystallographers work and collaborate today. Dorothy Hodgkin went on to Oxford and determined structures of biologically important small molecules as well as proteins, most notably insulin [3, 4]. Max Perutz arrived in Cambridge from Austria in 1936 and began his study of hemoglobin which led to its structure determination in 1959 [5]. Both Hodgkin and Perutz trained large numbers of crystallographers who set up laboratories around the world. John Kendrew arrived at Cambridge’s newly formed Medical Research Council Laboratory of Molecular Biology and determined the structure of myoglobin in 1957 [6, 7]. He went on to found the European Molecular Biology Laboratory. Kendrew, Perutz, and Hodgkin all received the Nobel Prize for their pioneering studies as did many other crystallographers [8]. In time, and perhaps not surprisingly, the structures of proteins began to emerge at a steady rate.

## Evolution of the Protein Data Bank

In the 1960s, crystallographers, computational biologists, and chemists became strongly interested in analyzing and visualizing these protein structures. However, the logistics of sharing these data was not straightforward. In the days before the Internet, it was necessary to send boxes of punched cards or magnetic tapes of coordinates through the mail. In 1971, a Cold Spring Harbor Symposium was held on “Structure and Function of Proteins at the Three-Dimensional Level” [9]. Leaders of the field described their exciting new results to a rapt audience. Among the attendees at the meeting was a prominent small molecule crystallographer named Walter Hamilton who together with Edgar Meyer was building a computer library of structures [10]. He offered to host the Protein Data Bank (PDB) at Brookhaven National Laboratory. Immediately after that meeting he flew to Cambridge and engaged Olga Kennard, who was the head of the Cambridge Crystallographic Data Centre, to collaborate on maintaining the archive. In October 1971, the PDB was announced in an article in *Nature New Biology* [11]. And so the PDB was launched with seven structures.

Over time, structural biology grew as new methods for protein production, crystallization, data collection, and structure analysis continued to be developed. As a consequence, the number of structures increased steadily, as did their complexity. In addition, NMR spectroscopy and electron cryo-microscopy began to be used for structure determination. The covers of *Science* and *Nature* were often adorned with beautiful examples of the structures of life.

In the 1980s there was an increasing demand to make deposition of published structures into the PDB mandatory. Articles and opinion pieces began to appear in which the structural biology community was challenged to make all their data publicly available [12]. The International Union of Crystallography (IUCr) established a committee whose task it was to create guidelines for the deposition of X-ray crystal structures. It was composed of leading people in the field, who worked very hard to define the exact content of a PDB deposition. At the same time, Fred Richards (Yale University) led a grassroots effort to encourage structural biologists to deposit their coordinates. This took the form of a petition that was signed by hundreds of distinguished scientists and led to the publication of guidelines in 1989 [13]. In time, virtually all journals that publish crystal structures of biomacromolecules made deposition into the PDB archive a requirement for publication. In the early 1990’s the National Institute of General Medical Sciences became the first funding agency to impose a similar requirement on all grantees that determined structures. At first, only deposition of coordinates was required. After continued community discussions, deposition of the experimental data that underpin structures (structure factor data for X-ray crystallographic studies; restraints for NMR studies) became mandatory in 2008. Deposition of chemical shift data for NMR structures became mandatory in 2010.

After Walter Hamilton’s untimely death in 1973, Tom Koetzle led the PDB at Brookhaven [14], and he was succeeded by Joel Sussman in 1994. In 1998, a call for proposals by the NSF resulted in the management of the PDB being taken over by the Research Collaboratory for Structural Bioinformatics (RCSB): a consortium formed by groups at Rutgers, The State University of New Jersey, the National Institute of Standards and Technology, and the University of California San Diego [15]. The RCSB PDB collaborated with data centers formed in Europe at the European Bioinformatics Institute (EMBL-EBI) and in Japan at Osaka University. In 2003, the Worldwide Protein Data Bank (wwPDB) was formed, uniting these centers to ensure that the PDB would remain a global, publicly available, and uniform archive [16, 17]. The wwPDB partners (RCSB PDB [15]; Protein Data Bank in Europe, PDBe [18]; Protein Data Bank Japan, PDBj [19]) developed clear guidelines and policies for data deposition and annotation. In 2006, the BioMagResBank (BMRB) archive of NMR experimental data joined the wwPDB [20, 21]. An Advisory Committee consisting of international leaders in structural biology meets annually to review the activities and policies of the wwPDB.

**Fig. 1:**
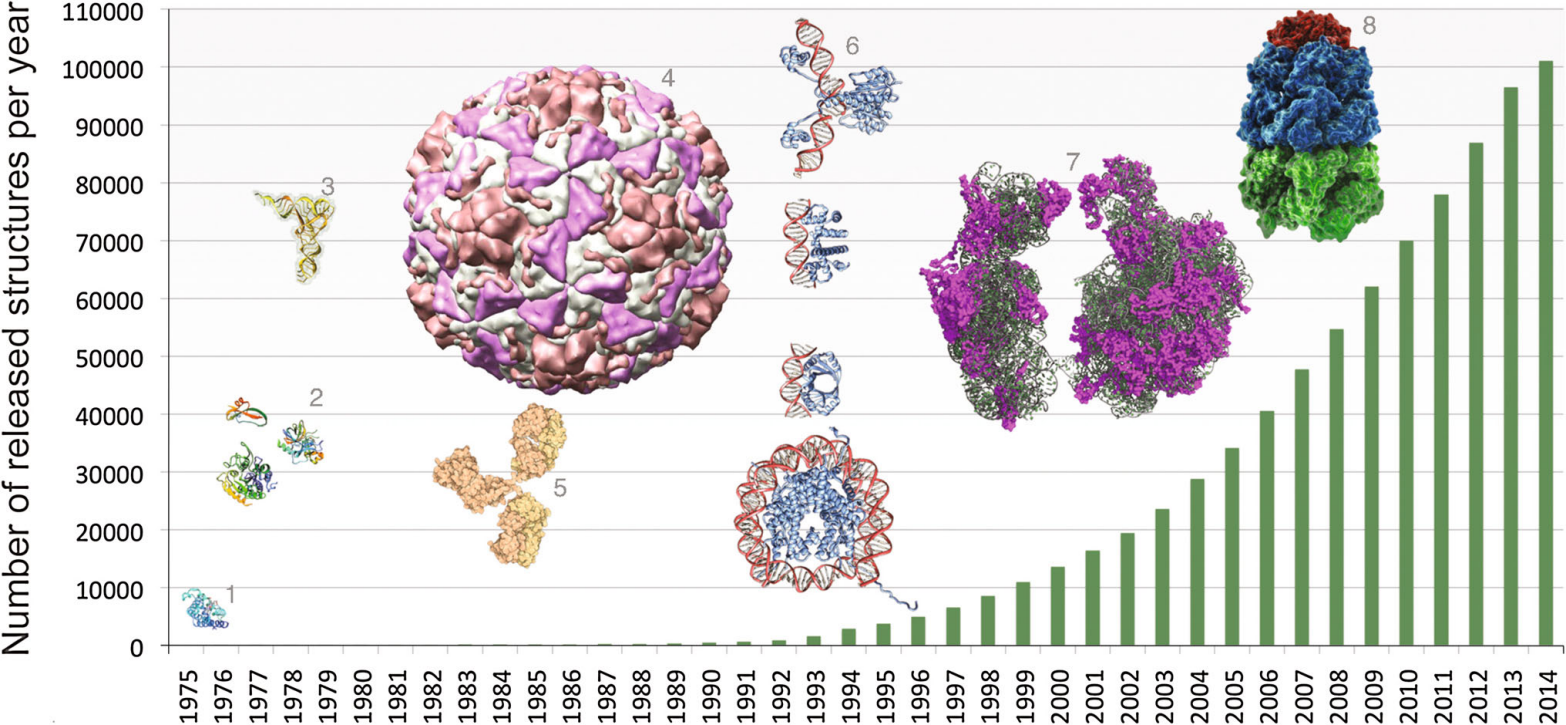
Growth of the PDB archive. Number of structures available in the PDB per year through June 18, 2014, with selected examples. Early structures included myoglobin (*1* PDB ID 1mbn [6, 7]), the first structure solved by X-ray crystallography, and small enzymes (*2* *top* 4pti [48], *bottom right* 2cha [49], *bottom left* 3cpa [50]). As technologies developed, the archive grew to host examples of tRNA (*3* 6tna [51]), viruses (*4* 4rhv [52]), antibodies (*5* 1igt [53]), protein-DNA complexes (*6* *top to bottom*, 1gdt [54], 1tro [55], 2bop [56], 1aoi [57]), ribosomes (*7* 1fjg, 1fka, 1ffk [58–60]), and chaperones (*8* 1aon [61])

In 2014, the PDB archive reached a milestone 100,000 entries (Fig. 1).

## Standards

In 1990, a committee appointed by the IUCr began a project to define standards for information exchange in macromolecular crystallography. Although the PDB file format that had been created in 1974 was widely used, restrictions on the number of atoms and polymer chains enforced by its 80-column fixed-field-width format meant that it could not accommodate large structures. The macromolecular Crystallographic Information File (mmCIF) was introduced in 1996 following a series of workshops and meetings [22]. Its dictionary contained more than 3,000 definitions of concepts covering the results of crystallographic experiments as well as the experiments themselves. mmCIF provides for typing and relationships among data items, and because it is self-defining, mmCIF is ideally suited for computational applications. In time, this dictionary came to include definitions for NMR and 3D cryo-Electron Microscopy (3DEM) and was renamed PDBx [23]. In spite of its advantages, it was not until 2011, at a seminal meeting at the EBI of senior wwPDB staff and key crystallographic software developers, that agreement was reached to use this format in all crystallographic software applications. Currently, discussions among major developers of NMR structure-determination and validation software are leading in a similar direction.

PDBx is now the “master format” for the PDB. Large structures such as ribosomes, which can only be represented in the old PDB file format by splitting a single structure into multiple entries, will be combined into single files and released later in 2014. A “round trip” is now possible whereby a coordinate file, which was produced by a refinement program, curated by wwPDB staff and released in the public archive, can then serve as input again for structure-refinement programs. The PDB format can be retired after the many programs that have depended on it are updated to accept and produce the more versatile PDBx format.

## Validation

Data deposited into the PDB are evaluated and processed to ensure that they are of the highest quality possible. Over time, the checks that are made have evolved considerably. Atom-naming, geometry and chemistry checks have been in place for many years. With the availability of mandatory experimental data such as structure factors, procedures were put in place to check the coordinates against the data.

In 2008, there were allegations that twelve structures published in journals and available in the PDB were based on fabricated data [24]. These structures, along with an ongoing concern for ensuring the quality of the data archive [25], motivated the wwPDB to convene an X-ray Validation Task Force (VTF) consisting of scientists with expert knowledge of crystallographic methods and validation procedures [26]. The X-ray VTF was charged with recommending best practices for validation that the wwPDB could then implement in its data-processing pipeline [27].

The VTF used a variety of methods to review the entire corpus of PDB data and a large set of validation statistics and methods. It made recommendations for how best to check the validity of the models and the experimental data and proposed a summary graphic to represent the overall quality of a structure relative to other PDB structures. Using these methods it was possible to identify the alleged fabricated structures. However, more importantly the validation methods help depositors and users alike to assess the quality of models, to identify unusual features, and to compare alternative models of the same molecule. They also provide important information to editors and referees of journals that require submission of the wwPDB validation reports with manuscripts describing new structures.

Following the success of the X-ray VTF, two more were established. The wwPDB NMR VTF of experts in NMR structure determination and validation reviewed structures in the PDB and made recommendations for validation [28]. A VTF of experts in 3DEM reviewed validation practices for maps in the Electron Microscopy Data Bank (EMDB) [29] and models in the PDB [30]. Their recommendations are the basis of an on-going research program to develop methods and software for 3DEM validation.

## Current capabilities and usage

At the time that the wwPDB was first formed different data deposition and processing systems were in operation at the different centers. These systems were reviewed in an effort to ensure that the results of data processing were the same no matter where the processing was carried out. Data among the sites were exchanged and reviewed. A few years ago, the wwPDB initiated an ambitious project to create a new Deposition and Annotation system (D&A) [31] in which the experience and existing software applications of all the sites formed the basis for creating a new, modular and more efficient system.

The resulting system, which for X-ray structures went into production-testing in early 2014, consists of a series of modules that allow for careful review of sequences, taxonomy, ligands, and the various other types of annotation that are part of a PDB deposition. Depositors submit data through a single portal. Assignment of the wwPDB deposition and annotation site takes into consideration geography and workload. Although each entry is checked and analyzed in greater detail than by the legacy systems, the processing is more automated and efficient, and the quality of the fully annotated structure files is higher.

A key component of the new system is the validation module, which embodies the recommendations of the X-ray VTF [27]. A detailed report is provided to the depositor that contains information about the quality of a structure and draws attention to any unusual features. A stand-alone server is also available so users can check a structure prior to deposition. The depositor can make the validation report available to journals, many of which now require these reports as part of the manuscript-review process. It is hoped that this careful assessment of models and data will have a positive impact on the overall quality of the structures that are published by journals and released in the PDB.

The PDB is one of the most widely used structural resources in biology. More than 400 million coordinate sets were downloaded in 2013 from the wwPDB partner sites. Both the utility and the uniformity of PDB data have enabled the development of other databases and data-related resources, including resources for drug discovery (for a review see [32]); resources focused on small molecules and ligands such as ChEMBL [33],  DrugBank [34], BindingDB [35], BindingMOAD [36], and PDBBind [37]; protein structure classification and annotation resources, such as CATH [38, 39], SCOP [40–42], and PDBsum [43, 44]; and focused, specialty annotation resources such as Protein Data Bank of Transmembrane Proteins (PDBTM) [45], ArchDB for functional loops in structures [46], and 3did for protein–protein interaction surfaces [47]. These resources are frequently compiled in the annual Database Issue of *Nucleic Acids Research.*

The users come from many areas of science including biology, chemistry, physics, and computer science. The PDB is also an important resource for teachers and students who want to learn about the molecules of life.

## Challenges

The PDB is managed by an international consortium of organizations, each of which must secure funding in different ways. The RCSB PDB receives funds from the NSF, NIH and DOE. PDBe receives funding from EMBL, the Wellcome Trust, NIH, EU, BBSRC and MRC. PDBj is funded by the Japan Science and Technology Agency, and BioMagResBank by NIGMS. Each site is on a different cycle and is reviewed every 3–5 years. This diversity of funding mechanisms is both a strength and a weakness. Since the probability of all sites losing all their funding is not high, it is likely that there will always be some funded centers to support the archive, although obviously not as efficiently as if all the sites were funded.

The availability of the new D&A system makes it possible for new centers to join the wwPDB in the future, thus helping to spread the workload of PDB curation, or assuming responsibility for a particular subset of structural data (an example is provided by the BMRB, which handles NMR-derived data not directly associated with the determination of an atomic model to be deposited in the PDB).

The face of structural biology is changing. Rather than one method being used to determine a single structure, it is becoming more common to use two or more methods and also to study structure at a variety of length scales. Integrative and multi-scale methods require coordination across disciplines and perhaps a different model for archiving the experimental data. In the next several years the wwPDB will be working with the various experimental and modeling communities to determine how best to manage the diversity of 3D structure data.

## Summary

In this perspective, we have outlined the evolution of the Protein Data Bank archive and have emphasized the key role that the community has played in helping to shape the resource and its management. Bernal’s ideal of collaborative science continues to be a driving force in structural biology.
